# On discontinuing treatment in schizophrenia: a clinical conundrum

**DOI:** 10.1038/s41537-016-0004-2

**Published:** 2017-01-12

**Authors:** Robin Emsley

**Affiliations:** Department of Psychiatry, Faculty of Medicine and Health Sciences, Stellenbosch University, PO Box 241, Tygerberg, 8000 Cape Town South Africa

We psychiatrists who treat individuals with schizophrenia spend much of our time encouraging our patients to take antipsychotic medication regularly. We do this because of concerns regarding the consequences of illness recurrence associated with treatment discontinuation. There is however a good deal of discomfort here, given the substantial side-effect burden accompanying such treatment, and the hope that some patients will, at some stage, be able to discontinue medication without experiencing illness recurrence. Also, there has been some suggestion that long-term antipsychotic treatment may actually contribute to poorer outcome^1,2^ and it has been speculated that the very high relapse rates reported after treatment discontinuation may be iatrogenic insofar as they represent withdrawal phenomena which could be physiological, psychological or a “supersensitivity psychosis” due to receptor adaptations to protracted dopamine D2 blockade.^3^ Finally, greater exposure to antipsychotic treatment has been associated with progressive brain volume reductions.^4^

But the reality is that, when treatment is discontinued, rates of illness recurrence are very high, even after a single psychotic episode,^5^ and there is no good evidence to support the supersensitivity psychosis hypothesis.^6^ Further, no successful discontinuation strategies have been described. Our ability to pre-empt illness recurrence is less than optimal. Early warning signs are not always apparent and rescue strategies not always effective.^7^ Finally, we are unable to predict which patients have a better chance of successfully discontinuing treatment—indeed, counterintuitively, patients who respond best to treatment may be particularly at risk of relapse.^8^ What is undisputed, is that relapse episodes may have harmful psychosocial and perhaps biological consequences.^7^

So where does this leave us? Efficacy of maintenance antipsychotic treatment for relapse-prevention is very well documented.^9^ As responsible clinicians we should advise all patients who have benefitted from antipsychotic treatment against treatment-discontinuation. How long should treatment continue? Indefinitely. There is no indication that a longer treatment period reduces risk of relapse.^7^ The side-effect burden should be managed by selecting the best tolerated antipsychotic, prescribing the lowest effective dose and instigating behavioural and other measures to reduce the risk of metabolic syndrome. A particular concern is the ongoing use of placebo in relapse-prevention clinical trials in schizophrenia where, contrary to our clinical role and to the principles of beneficence and clinical equipoise, clinicians are complicit in the decision to discontinue active treatment.^10^ Most importantly, there is an urgent need for better research exploring alternatives to indefinite treatment with currently available antipsychotic medication, and to investigate whether discontinuation strategies are feasible for any patients, at any stage of the illness.
